# Supplementary material for: Evolutionary game model of intellectual property pledge financing between technology-based SMEs and banks based on the EVCC
Source: PLoS One. 2025 Sep 5;20(9):e0331421. doi: 10.1371/journal.pone.0331421 (PMC12413005; doi:10.1371/journal.pone.0331421)
Supplement: Attachment — Submitted filename: Peer review - Game theory.docx [file pone.0331421.s001.docx]

Date: 2/21/2025

**Peer Review of the Manuscript:**

**Title: Evolutionary Game Model of Intellectual Property Pledge Financing Between Technology-based SMEs and Banks Based on the End-of-period Value Conversion Coefficient of Pledged Property.**

**1. Originality of Research**

The study presents an original research approach by integrating the end-of-period value conversion coefficient into an evolutionary game model for intellectual property (IP) pledge financing. The incorporation of this coefficient enriches the multi-agent game theory framework in this domain, making it a novel contribution.

**2. Novelty of Results**

The results reported in the manuscript appear to be unique and not published elsewhere. The manuscript offers new insights, particularly in analyzing the impact of key financial parameters on the strategic behavior of SMEs and banks. However, verification through a plagiarism check or comparison with existing literature would be necessary to confirm this.

**3. Technical Rigor and Methodological Standards**

- The study is methodologically sound, employing evolutionary game theory and numerical simulations to validate theoretical insights.
- The replication dynamic equations and stability analysis provide a robust theoretical foundation.
- The numerical simulations and parameter sensitivity analyses are thorough.
- The use of MATLAB for simulations enhances the study’s technical depth.

Potential Issue:

- The manuscript would benefit from further justification of parameter selection, particularly for variables that are subjectively assigned. High technical standard, but justification for parameter assignment could be strengthened.

**4. Validity of Conclusions**

- The conclusions are logically derived from the model and simulation results.
- The asymptotic stability analysis confirms the validity of the proposed strategies.
- The discussion section effectively interprets findings and compares them with prior studies.

Potential Issue:

- Some claims regarding the impact of parameters on strategic choices require further empirical validation beyond numerical simulations.

**5. Clarity and Language**

- The manuscript is written in clear and standard English.
- Technical terminologies are well-defined.
- However, some sections (e.g., mathematical formulations) could be made more readable by improving structural flow.

**6. Ethical Standards**

- The research does not involve human subjects or sensitive data.
- Ethical considerations related to research integrity are met.

**7. Reporting Standards & Data Availability**

- The study adheres to standard reporting guidelines in evolutionary game theory.
- However, explicit mention of data availability, including numerical simulation codes or underlying datasets, is lacking.
