# Supplementary material for: Evolutionary game model of intellectual property pledge financing between technology-based SMEs and banks based on the EVCC
Source: PLoS One. 2025 Sep 5;20(9):e0331421. doi: 10.1371/journal.pone.0331421 (PMC12413005; doi:10.1371/journal.pone.0331421)
Supplement: Attachment — Submitted filename: Academic editor comments PONE-D-24-56353.docx [file pone.0331421.s002.docx]

This paper presents a novel and methodologically sound evolutionary game model to analyse the strategic interactions between technology-based SMEs and banks in the context of intellectual property pledge financing. A key innovation is the introduction of the “end-of-period value conversion coefficient”, which attempts to model the willingness to perform financial obligations.

The theoretical framework is robust, and simulations are thorough. However, some key areas including parameter transparency, empirical contextualisation, presentation clarity, and data/code availability, require attention to meet PLOS ONE’s criteria for scientific validity, reporting transparency, and accessibility. Below are the principal areas where revisions are necessary to elevate the manuscript to a publishable standard:

The current title is too long and unwieldy, reducing accessibility and search engine optimisation. I suggest shortening and clarify the title in line with the reviewer recommendation

The manuscript uses subjectively assigned parameter values (e.g., penalty costs, return rates) without adequate empirical or theoretical backing. The author must consider add a Table or Appendix justifying parameter choices. As well as consider sensitivity ranges where values are hard to justify precisely.

The manuscript lacks information about data/code availability, which is required by PLOS ONE for transparency and reproducibility. The authors should upload the MATLAB code used for simulations to a repository.

The model is purely theoretical and simulation-based. Hence, some conclusions imply real-world policy implications without empirical backing. Therefore, the authors must clearly state in the Discussion that conclusions are theoretical and require empirical validation. As well as suggesting future work to calibrate the model using real data from Chinese IP pledge financing programs or banks.

Several critical equations are unnumbered or embedded in long, dense paragraphs. Therefore, authors must number all equations for ease of reference. And add brief intuitive explanations or summaries after each key equation set. Or perhaps consider using figures or diagrams to represent the game tree or model structure.

The figure captions lack detail, and variable meanings are not consistently clarified. Authors must ensure all axis labels are complete. In addition, expand captions to interpret the figures.

While the literature review is substantial, it could benefit from more recent international literature on IP financing or trust-based SME financing strategies. I suggest that author must incorporate newer references from 2022 onwards. Include Fintech and SME trust modelling, IP collateral valuation challenges and Game-theoretic applications in finance.

The manuscript refers to practical relevance but lacks concrete recommendations. In addition add a dedicated section (or at least a paragraph in the Conclusion) with practical implications which should include threshold values for policy intervention; How banks or governments might use the coefficient “k” and Incentive design for SME trustworthiness

Finally, the English is mostly clear, but some technical and mathematical sections are difficult to follow, shorter sentences and more active voice, rephrase dense mathematical paragraphs with bullets or boxed summaries where possible.
