# Supplementary material for: Evolutionary game model of intellectual property pledge financing between technology-based SMEs and banks based on the EVCC
Source: PLoS One. 2025 Sep 5;20(9):e0331421. doi: 10.1371/journal.pone.0331421 (PMC12413005; doi:10.1371/journal.pone.0331421)
Supplement: Attachment — Submitted filename: Response to Reviewers.doc [file pone.0331421.s004.doc]

List of Responses

Dear Editors and Reviewers:

Thank you for your letter and for the reviewers’ comments concerning our manuscript entitled “Evolutionary Game Model of Intellectual Property Pledge Financing Between Technology-based SMEs and Banks Based on the End-of-period Value Conversion Coefficient of Pledged Property” (ID: PONE-D-24-56353). Those comments are all valuable and very helpful for revising and improving our paper, as well as the important guiding significance to our research. We have studied comments carefully and have made correction which we hope meet with approval. Revised portions are marked in red in the paper. The main corrections in the paper and the responds to the reviewer’s comments are as follows:

Responds to the reviewer’s comments:

**Reviewer #1**:

1. Response to comment: (Strengthening the justification for parameter assignments.)

Response: Considering the Reviewer’s suggestion, we have added specific reference sources for each parameter assignment. See Section 3.1, paragraph 2 for details.

2. Response to comment: (Providing a data availability statement.)

Response: We have added a Data availability statement at the end of this paper according to the Reviewer’s suggestion.

3. Response to comment: (Improving readability in mathematical sections.)

Response: Considering the Reviewer’s suggestion, we have carefully modified the mathematical expressions in the mathematical sections. See Section 2.2, Section 2.3, Section 2.5 and Section 2.6 for details.

Special thanks to you for your good comments.

**Reviewer #2**:

1. Response to comment: (**Title Clarity:** Suggested revision: The title is long and dense. Consider rephrasing slightly for clarity and international accessibility. Proposed title: Evolutionary Game Model for Intellectual Property Pledge Financing: Evidence from End-of-Period Value Conversion in SME-Bank Interactions.)

Response: Considering the Reviewer’s suggestion, we have revised the manuscript title to: Evolutionary Game Model of Intellectual Property Pledge Financing Between Technology-based SMEs and Banks Based on the EVCC. See page 1 for details.

2. Response to comment: (**Abstract:** The abstract lacks numerical context for key parameters and simulation results. Suggested revision: Add specific numerical outcomes from the simulations (e.g. thresholds for the coefficient where ESS is achieved) to support claims. Example: “When the value conversion coefficient exceeds 1.15, cooperation becomes evolutionarily stable...”.)

Response: Considering the Reviewer’s suggestion, we have supplemented the threshold values of key parameters in the abstract. See Abstract for details.

3. Response to comment: (**Terminology Clarification:** The term "end-of-period value conversion coefficient" is introduced early but not clearly explained until later. Suggested revision: Define this term succinctly and mathematically in the abstract or early in the introduction, ideally with an equation or variable symbol (e.g. λ) for consistency.)

Response: According to the Reviewer’s suggestion, we have modified the title of Section 2.2 to: Definition of EVCC, and proposed Definition 1, using the symbol *k* to represent the end-of-period value conversion coefficient of pledged property (EVCC). See Section 2.2 for details.

4. Response to comment: (**Literature Review:** The literature section does not include recent empirical work on IP pledge valuation mechanisms. Suggested revision: Add 1–2 references from the last 2–3 years focusing on financial valuation of IP in emerging markets or fintech applications in SME lending.)

Response: Considering the Reviewer’s suggestion, we have added two new references: [20] and [23].

[20] Chen, Y., Yuan, J. L., Ren, K. J., et al. (2024). Analysis of the Evolution Game of Intellectual Property Pledge Financing from the Perspective of Blockchain. Brand and standardization, (05), 167-170.

[23] Ran, C. J., Zhang, Y. R., & Huang, W. J. (2025). Does artificial intelligence affect the credit risk of intellectual property pledge financing? - Evolutionary Game Analysis of Credit Risk of Intellectual Property Pledge Financing Based on Game Theory. Library construction, (03), 36-47+59. doi: https://doi.org/10.19764/j.cnki.tsgjs.20250614

5. Response to comment: (**Figure Annotations:** Figures lack detailed captions and axis labels are not always intuitive (e.g. "repayment willingness" as a Y-axis). Suggested revision: Include full mathematical labels (e.g., "Probability of strategy adoption: Repayment (p)") and clearly describe what the curves represent in the caption.)

Response: According to the Reviewer’s suggestion, we have added a detailed description of the horizontal axis and the vertical axis for each figure in the text, and explained the meaning of the curve in the figure. See Section 3.2.1 and Section 3.2.2 for details.

6. Response to comment: (**Numerical Simulation Parameters:** Parameter values for simulation (e.g., payoff matrix elements, initial population proportions) are buried in the text. Suggested revision: Add a dedicated table summarizing all input parameters for the numerical simulation, with justifications or citations if applicable.)

Response: Considering the Reviewer’s suggestion, we have summarized all parameter assignments in Table 4, and added specific reference sources for each parameter assignment. See Section 3.1 for details.

7. Response to comment: (**Grammar and Style:** Several sentences contain grammatical errors or awkward phrasing. Examples & Fixes: “To improve the market efficiency of intellectual property pledge financing...” → “This study aims to improve the efficiency of IP pledge financing...”; “...construct the end-of-period value conversion coefficient of pledged property to measure the comparative relationship...” → “...define a conversion coefficient to measure the ratio between pledged IP value at maturity and total loan repayment.”)

Response: According to the Reviewer’s suggestion, we have carefully corrected the grammar errors in both the abstract and the main text. The sections with significant changes include: Abstract, Section 1, Section 2.2, Section 2.3, Section 2.5, Section 2.6, Section 3.2.1, Section 3.2.2, Section 4, and Section 5.

8. Response to comment: (**Policy Implications Section****:** The policy section is too generic. Suggested revision: Include specific recommendations, such as “Development of national IP valuation guidelines” or “Incentives for banks offering IP-backed financing.”)

Response: Considering the Reviewer’s suggestion, we have added a natural paragraph in Section 5 to express policy recommendations. See Section 5, penultimate paragraph.

9. Response to comment: (**Mathematical Consistency:** Equation numbers are inconsistent and sometimes missing. Suggested revision: Ensure all key equations are numbered and referenced in the text for easy traceability.)

Response: Considering the Reviewer’s suggestion, we have put the unnumbered formula in Section 2.2 into the text, to maintain the continuity and consistency of the formula number. See Section 2.2 for details.

Special thanks to you for your good comments.

**Reviewer #3**:

1. Response to comment: (In the second line of the first paragraph, SMEs are not specifically explained.)

Response: Considering the Reviewer’s suggestion, we have supplemented the definition of technology-based SMEs in Section 1, paragraph 1. See Section 1, paragraph 1 for details.

2. Response to comment: (The article uses past and past perfect tenses, which may indicate machine translation.)

Response: According to the Reviewer’s suggestion, we have carefully corrected the grammar errors in both the abstract and the main text. The sections with significant changes include: Abstract, Section 1, Section 2.2, Section 2.3, Section 2.5, Section 2.6, Section 3.2.1, Section 3.2.2, Section 4, and Section 5.

3. Response to comment: (The references include Chinese literature, and I suggest changing them to English.)

Response: Considering the Reviewer’s suggestion, we have carefully proofread all references to ensure that all references are in English.

Special thanks to you for your good comments.

**Other changes:**

1. We have removed the previously weakly related reference [16], and added the reference [33] in the parameter assignment.

2. Due to the addition of 3 references and deletion of 1 reference, we have adjusted the reference numbers.

3. We have added a “Funding State” and a “Conflicts of Interest” at the end of this paper.

We tried our best to improve the manuscript and made some changes in the manuscript. These changes will not influence the content and framework of the paper. And here we did not list the changes but marked in red in revised paper.

We appreciate for Editors/Reviewers’ warm work earnestly, and hope that the correction will meet with approval.

Once again, thank you very much for your comments and suggestions.
